# Factors Associated with Selection of Denture Adhesive Type: A Cross-Sectional Survey

**DOI:** 10.3390/jcm12030873

**Published:** 2023-01-21

**Authors:** Kohei Yamaguchi, Yohei Hama, Hitomi Soeda, Keita Hatano, Mitsuzumi Okada, Ryota Futatsuya, Shunsuke Minakuchi

**Affiliations:** Department of Gerodontology and Oral Rehabilitation, Graduate School of Medical and Dental Sciences, Tokyo Medical and Dental University, Tokyo 113-8549, Japan

**Keywords:** denture adhesive, home-liner, residual ridge condition, duration of denture use

## Abstract

The type of denture adhesive, cream or home-liner, chosen by regular denture adhesive users and oral conditions contributing to this selection require elucidation. The factors associated with denture adhesive selection were investigated through a face-to-face survey on oral and denture conditions. Age, sex, oral moisture, masticatory performance, retention and stability of the removable denture, ridge shape, mucosal thickness, and duration of denture use were examined in cream and home-liner-type denture adhesive users who did not regularly visit a dentist. Univariate analysis and multivariate analyses were performed. There were 38 and 40 cream-type and home-liner-type adhesive users, respectively. The type of denture adhesive was significantly associated with the oral moisture value, retention, ridge shape, mucosal thickness, and duration of denture use in univariate analyses. The residual ridge conditions with large factor loadings for ridge shape and mucosal thickness and duration of denture use were significantly related to the denture adhesive selection in multivariate logistic analysis. The residual ridge conditions and duration of denture use were significant factors in the selection of cream- and home-liner-type denture adhesives. These results can provide appropriate guidance based on the adhesives patients without dental supervision are more likely to choose.

## 1. Introduction

Japan has become a super-aged society, and the number of older adults is expected to increase in the future [1]. Although the number of remaining teeth in older adults has been increasing [2], previous surveys in Japan reported that the removable denture usage rate was approximately 30% among 1,875 older adults living in a community with an average age of 66.7 years [3] and approximately 40% among 1272 people with an average age of 69.7 years [4]. These studies indicate that there are still many people who use removable dentures. Furthermore, various types of denture adhesives are known, including cream, home-liner, powder, and sheet types. Cream, powder, and sheet types are categorized as narrowly defined denture adhesives. A web-based survey of 1,470 denture wearers in Japan reported that 21.6% of participants used denture adhesives, of which 66.7% used the cream type, followed by the liner type (23.3%) [5]. Another web-based study has reported that 23.3% of denture wearers use denture adhesives, with 79.7% using the cream type and 16.2% using the liner type [6]. Thus, users of these two types of denture adhesives exist to some extent in Japan.

Denture adhesives are sold at drug shops in Japan, and anyone can buy and use denture adhesives on their own initiative. The cream type is classified as a denture adhesive in the narrow sense of the term and exhibits adhesive properties when mixed with saliva between the mucous surface of the denture base and subfloor mucosa. It has high fluidity and spreads thinly; therefore, it is less likely to cause changes in the occlusal vertical dimension. The thinner the denture adhesive, the greater the adhesion strength [7]. Generally, the cream type must be cleaned at daily intervals and should not be used for more than one day. On the other hand, the home-liner type is a highly viscous material that fills the gap between the denture base mucosa surface and the subfloor mucosa. The home-liner-type adhesive can be used for a few days, but there is no published evidence stating this.

The effects of cream-type denture adhesives include improvement in the stability of well-fitting dentures [8], masticatory performance [9], patient satisfaction, mandibular movement during chewing [10], and denture retention [11]. Risks of using denture adhesives include residual ridge resorption, denture stomatitis, *Candida* infection, and oral flora imbalance [12]. Although there is little longitudinal research, one study has reported that there is no problem with microorganisms for a 2-month period of use [13]. There are currently no long-term progress reports in the literature. It has also been shown that the home-liner-type denture adhesive improves patient satisfaction and masticatory performance by filling the gap between the denture and mucosa due to natural bone resorption and providing a cushioning effect [14,15,16]. The risk of using it includes occlusal misalignment, which may cause residual ridge resorption [17,18]. Therefore, since each denture adhesive has its particular advantages and disadvantages, dentists must instruct patients to use denture adhesives correctly. Dentists need to understand the denture adhesive more likely to be selected by people and the conditions under which this selection is performed to provide guidance according to the tendency of selection. 

A previous study, a web-based survey of 1470 denture wearers, did not identify any significant factors related to the type of denture adhesive selection [5]. In another web-based survey of denture adhesive use, 59.9% of respondents chose “Saw in the pharmacy/drugstore/store” and 19.3% chose “Saw an advertisement” as their reason for choosing a denture adhesive product [6]. This is only a subjective answer by the users, and thus, objective factors, of which the respondents themselves may be unaware, were not investigated. Therefore, the present study was conducted to examine the objective factors related to denture adhesive selection by conducting a face-to-face survey of oral and denture conditions. The null hypothesis of this study was that oral and denture conditions were not related to the selection of denture adhesive type. The purpose of this study was to identify the factors involved in the selection of cream- and home-liner-type denture adhesives among individuals who use denture adhesives on a daily basis.

## 2. Materials and Methods

### 2.1. Participant Characteristics

Participants in this study were recruited via e-mail sent to those registered with the research companies. The inclusion criteria were as follows: age 50 years or older, use of a cream or home-liner type of denture adhesives at least once a week, more than 6 months of use, no regular visits to the dentist, not currently undergoing dental treatment, and consent to face-to-face measurement. Participants in Eichner classification groups A and B1 and those using intermediary defect dentures were excluded. Written consent was obtained from all participants at the time of measurement. This study was approved by the Ethics Review Committee of Tokyo Medical and Dental University (D2018-057).

### 2.2. Measurements

#### 2.2.1. Oral Moisture

Using an oral moisture checker (Mucus; Life Co., Ltd., Saitama, Japan), the mucosal wetness of the tongue was measured thrice at the center of the tongue, and the median score was used as the “mucus value” to evaluate the oral moisture [19].

#### 2.2.2. Masticatory Performance

Color-changeable chewing gum (Mastication Check Gum; Lotte Co., Ltd., Tokyo, Japan) was used to evaluate the masticatory performance without denture adhesives. The gum was chewed 100 times, with one chew per second, without specifying the chewing side or using denture adhesives. The chewed gum was pressed using two glass slabs to a 1.5 mm thickness. The L^*^, a^*^, and b^*^ values (CIELAB color system) of the sample were measured using a colorimeter (CR13; Konica Minolta Sensing, Tokyo, Japan) at the center and at 3 mm to the top, bottom, left, and right of the center, and the average value was obtained. The masticatory ability evaluation value (MPIG) was calculated using ΔE [20,21].

(1) $$\Delta E=\sqrt{{\left(L^{*}-72.3\right)}^{2}+{\left(a^{*}-\left(-14.9\right)\right)}^{2}+{\left(b^{*}-33.0\right)}^{2}}$$

(2) $$\mathrm{MPIG}=\frac{1}{9.55\times 10^{-3}}\ln\left(\frac{-2.85\times 10^{7}}{\Delta E-73.2}-1\right)-1.35\times 10^{3}$$

(L^*^, a^*^, b^*^: coordinates in CIELAB color space).

A higher MPIG indicates higher masticatory performance.

#### 2.2.3. Retention and Stability

Denture retention and stability were evaluated according to Kapur’s classification [22]. Kapur scored the denture retention, stability, and ridge shape. The retention criterion is scored as follows: 0, no retention (when a denture is seated in its place, it displaces itself); 1, minimum retention (when a denture offers slight resistance to vertical pull and little or no resistance to lateral force); 2, moderate retention (when a denture offers moderate resistance to lateral force); 3, good retention (when a denture offers maximum resistance to vertical pull and sufficient resistance to lateral force). Scores of 0 and 1 were defined as “not enough” and 2 and 3 as “good”. The stability criterion is scored as follows: 0, no stability (when a denture base demonstrates extreme rocking on its supporting structures under pressure); 1, some stability (when a denture base demonstrates moderate rocking on its supporting structures under pressure); 2, sufficient stability (when a denture base demonstrates slight or no rocking on its supporting structures under pressure). Scores of 0 and 1 were defined as “not enough” and 2 as “good”.

#### 2.2.4. Residual Ridge and Mucosal Thickness

The residual ridge was evaluated according to Kapur’s classification [22]. The ridge shape was classified as V-shaped, flat, depressed, or U-shaped. Mucosal thickness was classified as thin, normal, or thick by manipulation. These were evaluated by one prosthodontist with at least 10 years of clinical experience.

#### 2.2.5. Duration of Denture Use

The questionnaire asked about the duration of denture use (more or less than three years).

### 2.3. Statistical Analysis

The Shapiro–Wilk test was performed to evaluate the normality of the data. To check the differences between the two groups of cream- and home-liner-type users, Wilcoxon’s rank-sum test was performed for age, and chi-squared tests were performed for sex and denture type.

#### 2.3.1. Univariate Analysis

Logistic regression analysis was performed with the type of denture adhesive as the objective variable and mucus value and MPIG as independent variables.

A chi-squared test was performed with the type of denture adhesive as the objective variable and the ridge shape, mucosal thickness, retention, stability, and duration of denture use were used as independent variables.

#### 2.3.2. Multivariate Analysis

Exploratory factor analysis was performed to consolidate variables. Factor analysis was performed on the correlation matrix, and the maximum likelihood method was used. Factor loadings were adjusted using varimax rotation to make the analysis easier. To determine the number of factors, the Kaiser–Guttman criterion was used up to a factor with an eigenvalue greater than 1 and selected factors that contained items with an absolute factor loading of at least 0.3. Multivariate logistic regression analysis was performed on three different factors resulting from the exploratory factor analysis, with age and sex as independent variables and the denture adhesive type as the dependent variable.

All statistical significance levels were set at *p* = 0.05, and the statistical software JMP8.0 (SAS Institute, Cary, NC, USA) was used.

## 3. Results

### 3.1. Characteristics of Participants

Among the respondents, 88 registrants agreed to participate and be measured face-to-face, and 10 participants were excluded because they had used denture adhesives for less than 6 months. The participants’ characteristics are listed in Table 1. No significant differences were found in age, sex, type of denture, or masticatory performance between the types of denture adhesives. Significant differences were found only in oral moisture.

#### 3.1.1. Univariate Analysis

Table 2 shows the logistic regression analysis results with the denture adhesive type as the objective variable and mucus value and MPIG as independent variables. Significant associations were found for the mucus value. 

The results of the chi-squared test are shown in Table 3. Significant differences were found in denture retention, ridge shape, mucosal thickness, and duration of denture use.

#### 3.1.2. Exploratory Factor Analysis

Table 4 shows the factor loadings resulting from exploratory factor analysis of the 7 univariate independent variables. These were aggregated into 3 factors with eigenvalues greater than 1, with a cumulative contribution of 62.6%. The items with an absolute factor loading of 0.3 or greater were the retention of denture and stability of denture (Factor 1), ridge shape and mucosal thickness (Factor 2), and duration of denture use (Factor 3).

#### 3.1.3. Multivariate Analysis

A multivariate logistic regression analysis was performed on Factors 1, 2, and 3, with age and sex as the independent variables and denture adhesive type as the dependent variable (Table 5). Significant associations were found between Factors 2 and 3.

## 4. Discussion

This is the first study to examine the factors associated with the selection of cream- and home-liner denture adhesives by evaluating the oral and denture conditions among those who used denture adhesives on a daily basis. This study found significant factors associated with the selection of the type of denture adhesive, and these results are useful for predicting the adhesive a patient is likely to choose without supervision and in providing appropriate guidance.

Denture adhesives should be used only for the minimally required period of time under the supervision of a dentist [12]. The choice of denture adhesive for patients under supervision is likely to be heavily influenced by the dentist’s instructions. Therefore, patients who do not see a dentist regularly and who choose their own denture adhesives should be targeted to examine the factors associated with the selection of the type of denture adhesive.

The results of this study may also be useful in educating patients who visit the dental clinic regarding denture adhesives as it will allow one to predict the denture adhesive they may choose in the future. Moreover, in a previous web survey, 28.5% of denture adhesive users had not visited a dentist for more than 1 year [5]. This group of non-dentist visiting denture adhesive users should not be ignored, and it would be useful to know the tendencies related to their selection of denture adhesive.

In this study, those who had started using denture adhesives for less than 6 months were excluded. It has been reported that the most common reasons for choosing denture adhesives were “Saw the product in the pharmacy/drugstore/store” (59.9%) followed by “Saw an advertisement” (19.3%) [6]. However, it can be assumed that those who are still using a denture adhesive after 6 months are aware of its effectiveness, regardless of the subjective reason for their initial choice of adhesive. Therefore, it is meaningful to examine the relationship between the type of adhesive the individual chooses to use and objective evaluation in this study.

As mentioned above, a previous study using a web-based questionnaire failed to identify factors related to the selection of denture adhesive type [5]. This in-person study focused on oral and denture conditions. Considering the different characteristics of the cream or home-liner type, the following measurement items were selected as possible factors related to the selection of denture adhesives. First, considering that the purpose of denture adhesives is to improve retention the oral moisture value was measured, which is related to denture retention [23]. One of the main purposes of a prosthesis is to improve the masticatory function; thus, the masticatory performance without denture adhesives was evaluated as a criterion of denture quality. As another evaluation for dentures, Kapur’s classification [22] was used, which is used in denture adhesive research to evaluate retention and stability [16]. Furthermore, as denture-supporting tissue is associated with the selection of the type of denture adhesives, the residual ridge shape and mucosal thickness were evaluated with reference to Kapur’s classification. Finally, dentures may become incompatible over time [24,25] due to residual ridge resorption and incongruity in an occlusal relationship. Therefore, the duration of denture use of the present denture was considered in a comprehensive evaluation of dentures.

Logistic regression analysis showed that participants with higher mucus values are more likely to use the cream-type adhesive (Table 2). The cream-type adhesive would be suitable for those with a high oral moisture content as it mixes with saliva to increase the viscosity. Pearson’s chi-squared test showed that the usage rate of cream-type denture adhesive users was significantly higher for those with a U-shaped ridge, normal mucosal thickness, not enough denture retention, and more than 3 years of denture use (Table 3). It is possible that participants with unfavorable ridge shapes or thin mucosa selected viscoelastic home-liner-type adhesives to prevent pain. It is also natural that they would choose cream-type adhesives with viscous properties when the retention is insufficient. The results indicated that cream-type denture adhesives were more likely to be used by long-term denture users.

This study included 38 cream-type and 40 home-liner-type users. The sample size was originally planned to be approximately 70 subjects in each group, assuming 7 items were to be used in the multivariate analysis. However, the COVID-19 pandemic made it difficult to secure the planned sample size. Therefore, exploratory factor analysis was conducted to consolidate the independent variables.

Using exploratory factor analysis, the factors were aggregated into three, and each factor was interpreted based on the number of factor loadings. Factor 1 was referred to as “retention and stability” because of the large factor loadings for the retention and stability of dentures. In other words, Factor 1 is high when the retention and stability of dentures are good. Factor 2 was named the “residual ridge condition” because of the large factor loadings of the mucosal thickness and the ridge shape. Factor 2 is high when the mucosal thickness is thick and the ridge shape is good. Factor 3 was the “duration of denture use” because it has a large factor loading only for the duration of denture use. Factor 3 was high for less than 3 years of denture use.

In the multivariate analysis, the residual ridge condition and duration of denture use were significant. In other words, the better the residual ridge condition and the longer the duration of denture use, the greater the probability of using the cream type. After adjusting for age, sex, and denture condition, the results of the multivariate analysis were similar to those of the univariate analysis for residual ridge condition. Unfortunately, no studies have directly compared cream-type and home-liner-type adhesives and evaluated the differences in their properties and effects. Therefore, we have discussed studies that independently evaluated creams and home-liners. A previous study reported that the use of liner-type denture adhesive greatly improved the retention, masticatory ability, self-confidence in social activities, and satisfaction of participants, especially among those with poor supporting tissues (Kapur index) and those who reported poor retention of their previous dentures [16]. This result is one reason why the use of liner-type denture adhesives was more likely among those with poor ridge conditions in the present study. From a clinical standpoint, the choice of viscoelastic home-liner-type adhesives for sharp ridges and thin mucosa may make sense.

In addition, the probability of usage of a cream-type adhesive was higher when Factor 3 was small. No study has examined the effect of cream-type adhesives on the duration of denture use; however, cream-type adhesives may have an improvement effect, at least from the patient’s subjective point of view, on dentures in long-term users. Of course, continued use of denture adhesives in denture wearers without dentist supervision is not recommended, but dentists should be aware that long-term denture wearers tend to choose creams when using denture adhesives and should instruct them to go to a dental clinic first instead of immediately choosing a cream-type denture adhesive.

This study has several limitations. First, sampling bias is possible. The participants were selected from those who self-registered on a website as a sample of the population that uses denture adhesives on a daily basis. Participation was therefore limited to those who could access the internet. As a result, the participants were independent elderly people who had high information literacy skills and did not have severe problems related to activities of daily living. In addition, the participants were those who did not currently make regular dental visits and may differ from those under dental supervision in terms of health literacy and other factors. Second, the study did not assess the subjective reasons for choosing the denture adhesive type. The price of denture adhesives may also be included as a reason for selection. Although this study focused on objective measures to examine factors associated with the selection of the type of denture adhesive, it would have been more meaningful and applicable to a clinical setting if the subjective reasons for denture adhesive selection by each adhesive user had been also included. Finally, this study did not examine differences in effects or side effects. These factors are also important to understand and need to be examined in the future. 

From the above, the null hypothesis was rejected, and it was shown that denture conditions such as retention, stability, and duration of denture use were related to the selection of denture adhesive. In other words, denture wearers with a poor residual ridge condition were more likely to select a home liner, and denture wearers with a long duration of denture use were more likely to select a cream type. These results are significant for providing guidance regarding the adhesive a patient is likely to choose when they suspend dental supervision in the future or when they are not currently under dental supervision. Further research will be conducted to determine the reasons why the residual ridge condition and duration of denture use are related to the selection of the type of denture adhesives, including subjective factors.

## Figures and Tables

**Table 1:** Characteristics of participants.

	Cream	Home-Liner	*p*-Value
N	38	40	
Mean age, years (SD)	70.5 (5.9)	69.5 (8.0)	0.95 ^a^
Sex			
Male	29	23	0.08 ^b^
Female	9	17	
Type of denture			
Partial	14	15	0.95 ^b^
Complete	24	25	
Oral moisture	27.5 [26, 28.9]	26.9 [26, 27.9]	**0.04** ^a^
Masticatory performance	104 [83.4, 122]	110 [90.6, 132]	0.29 ^a^

^a^: Wilcoxon rank sum test; ^b^: Pearson’s chi-squared test. SD, standard deviation. If either the upper or lower arch was edentulous, the patient was categorized as having a complete denture. Oral moisture and masticatory performance data are presented as median (25th percentile, 75th percentile). Bold faces denote significance (*p* < 0.05).

**Table 2:** Logistic regression analysis with the type of denture adhesive as a dependent variable.

Independent Variables	OR [95%CI]	*p*-Value
Oral moisture	1.31 [1.02–1.76]	**0.03**
Masticatory performance	0.99 [0.98–1.01]	0.35

OR [95%CI]: odds ratio (95% confidence interval). Boldface denotes significance (*p* < 0.05).

**Table 3:** Pearson’s chi-squared test with denture adhesive as a dependent variable.

Independent Variables	Cream	Home-Liner	Cream Ratio	OR [95%CI]	*p*-Value
Retention of denture					
Not enough	16	7	69.6%	0.29 [0.10–0.82]	**0.01**
Good	22	33	40.0%		
Stability of denture					
Not enough	24	22	52.2%	0.71 [0.29–1.77]	0.46
Good	14	18	43.8%		
Ridge shape					
V-shaped, flat, depressed	13	26	33.3%	3.57 [1.40–9.08]	**<0.01**
U-shaped	25	14	64.1%		
Mucosal thickness					
Thin	1	17	5.6%	27.3 [3.41–220]	**<0.01**
Normal, thick	37	23	61.7%		
Duration of denture use					
>3 years	36	25	59.0%	0.09 [0.02–0.44]	**<0.01**
<3 years	2	15	11.8%		

OR [95% CI]: odds ratio (95% confidence interval); Boldface denotes significance (*p* < 0.05).

**Table 4:** Exploratory factor analysis.

Independent Variables.	Factor Loadings after Rotation		
	Factor 1	Factor 2	Factor 3
Oral moisture	0.18	0.25	−0.07
Masticatory performance	0.02	0.01	0.27
Retention of denture	**0.44**	−0.19	0.06
Stability of denture	**1.00**	0.07	0.06
Ridge shape	−0.05	**0.64**	−0.01
Mucosal thickness	−0.03	**0.76**	−0.06
Duration of denture use	0.09	−0.22	**0.97**

Boldface denotes an absolute value > 0.3.

**Table 5:** Multivariate adjusted logistic regression analyses with denture adhesive type as the dependent variable.

Independent Variables	OR [95% CI]	*p*-Value
Factor 1 (retention and stability)	0.72 [0.39–1.29]	0.27
Factor 2 (residual ridge condition)	5.50 [2.40–15.8]	**<0.01**
Factor 3 (duration of denture use)	0.40 [0.18–0.76]	**<0.01**
Age	1.01 [0.92–1.11]	0.86
Sex (male/female)	1.50 [0.42–5.59]	0.51

Boldface denotes significance (*p* < 0.05). OR [95% CI]: odds ratio (95% confidence interval).

## Data Availability

Not applicable.
